# Draft Genome Sequence of *Pseudomonas chlororaphis* YL-1, a Biocontrol Strain Suppressing Plant Microbial Pathogens

**DOI:** 10.1128/genomeA.01225-13

**Published:** 2014-01-30

**Authors:** Youzhou Liu, Shi-En Lu, Sonya M. Baird, Junqing Qiao, Yan Du

**Affiliations:** a Institute of Plant Protection, Jiangsu Academy of Agricultural Sciences, Nanjing, China; b Department of Biochemistry, Molecular Biology, Entomology and Plant Pathology, Mississippi State University, Mississippi State, Mississippi, USA

## Abstract

*Pseudomonas chlororaphis* YL-1 was isolated from soybean root tips and showed a broad range of antagonistic activities to microbial plant pathogens. Here, we report the high-quality draft genome sequence of YL-1, which consists of a chromosome with an estimated size of 6.8 Mb with a G+C value of 63.09%.

## GENOME ANNOUNCEMENT

Compared with chemical pesticides, biological control represents a safer, more environmentally friendly approach to managing plant pathogens (1). *Pseudomonas* species are particularly suitable to be used as agricultural biocontrol agents because they can produce large amounts of metabolites to protect plants from pathogens (2–4). Most research of *Pseudomonas chlororaphis* has focused on its antifungal activities to phytopathogens and has scarcely addressed antibacterial activities (5–10). However, *P. chlororaphis* YL-1, which was isolated from soybean root tips, can suppress both fungal pathogens and bacterial pathogens of agricultural significance *in vitro*, such as *Burkholderia glumae*, *Clavibacter michiganesis*, *Erwinia amylovora*, *Rhizoctonia solani*, *Sclerotinia sclerotiorum*, and *Fusarium oxysporum*. Here, we analyzed the genome sequence of strain YL-1 to explore its features as a potential biocontrol agent.

The genome of *P. chlororaphis* YL-1 was sequenced using the Illumina/Solexa HiSeq 2000 technology at the Shanghai Majorbio Bio-pharm Technology Co., Ltd. (Shanghai, China), which generated 101-bp paired-end reads. We obtained approximately 12.6 million reads for assembly after the low-quality reads were filtered out. The whole genome was *de novo* assembled into 89 contigs (*N*_50_, 251,854 bp) and rearranged into 82 scaffolds using SOAPdenovo (11). Comparisons between contigs and scaffolds were then made using Glimmer 3.0. Because the circular genome sequence of *P. chlororaphis* has not been completed until now, the circular genome of *P. fluorescens* Pf0-1 (NC_007492.2) was used as a reference. Annotation was performed with the Rapid Annotations using Subsystems Technology (RAST) server version 4.0 (12), generating 6,368 candidate protein-encoding genes.

The draft genome of strain YL-1 consists of 6,801,259 bases with an average of 179-fold coverage and 63.09% G+C content. A cursory search of the genome sequence revealed the presence of gene clusters putatively involved in the synthesis of phenazine (PHZ) (13–16), pyrrolnitrin (PRN) (17, 18), and hydrogen cyanide (HCN) (19), which play important roles in biological activities. Interestingly, a putative *fit* (*ABCDEFGH*) operon that might be responsible for the synthesis of Fit insect toxin (20) was identified in the genome. Additionally, some genes which are involved in the synthesis of pyoverdine and achromobactin (21, 22) were also identified. There are multiple genes encoding antioxidative stress proteins, such as 5 catalases, 17 peroxidaes, 2 superoxide dismutases, and 2 transcription factors, SoxR and OxyR (23, 24). Moreover, genes encoding pyrroloquinoline quinine (*pqqBCDEF*) were also identified in the genome (25–27). A more specific analysis of strain *P. chlororaphis* YL-1 will be carried out in future research.

### Nucleotide sequence accession number.

The draft genome sequence of *P. chlororaphis* YL-1 has been deposited at DDBJ/EMBL/GenBank under the accession number AWWJ00000000. The version described in this paper is the first version.

## References

[1] Selin C Habibian R Poritsanos N Athukorala SN Fernando D de Kievit TR 2010 Phenazines are not essential for *Pseudomonas chlororaphis* PA23 biocontrol of *Sclerotinia sclerotiorum*, but do play a role in biofilm formation. FEMS Microbiol. Ecol. 71:73–83. 10.1111/j.1574-6941.2009.00792.x 19889032

[2] Liu H He Y Jiang H Peng H Huang X Zhang X Thomashow L Xu Y 2007 Characterization of a phenazine-producing strain *Pseudomonas chlororaphis* GP72 with broad-spectrum antifungal activity from green pepper rhizosphere. Curr. Microbiol. 54:302–306. 10.1007/s00284-006-0444-4 17334842

[3] Lugtenberg B Kamilova F 2009 Plant-growth-promoting rhizobacteria. Annu. Rev. Microbiol. 63:541–556. 10.1146/annurev.micro.62.081307.162918 19575558

[4] Mendes R Kruijt M de Bruijn I Dekkers E van der Voort M Schneider JH Piceno YM DeSantis TZ Andersen GL Bakker PA Raaijmakers JM 2011 Deciphering the rhizosphere microbiome for disease-suppressive bacteria. Science 332:1097–1100. 10.1126/science.1203980 21551032

[5] Haas D Défago G 2005 Biological control of soil-borne pathogens by fluorescent *pseudomonads*. Nat. Rev. Microbiol. 3:307–319. 10.1038/nrmicro1129 15759041

[6] Bardas GA Lagopodi AL Kadoglidou K Tzavella-Klonari K 2009 Biological control of three *Colletotrichum lindemuthianum* races using *Pseudomonas chlororaphis* PCL1391 and *Pseudomonas fluorescens* WCS365. Biol. Contr. 49:139–145. 10.1016/j.biocontrol.2009.01.012

[7] Maddula VS Pierson EA Pierson LS 3rd 2008 Altering the ratio of phenazines in *Pseudomonas chlororaphis* (*aureofaciens*) strain 30-84: effects on biofilm formation and pathogen inhibition. J. Bacteriol. 190:2759–2766. 10.1128/JB.01587-07 18263718 PMC2293254

[8] Park JY Oh SA Anderson AJ Neiswender J Kim JC Kim YC 2011 Production of the antifungal compounds phenazine and pyrrolnitrin from *Pseudomonas chlororaphis* O6 is differentially regulated by glucose. Lett. Appl. Microbiol. 52:532–537. 10.1111/j.1472-765X.2011.03036.x 21362001

[9] Fernando WGD Zhang Y Nakkeeran S Savchuk S 2007 Biological control of *Sclerotinia sclerotiorum* (Lib.) de Bary by *Pseudomonas* and *Bacillus* species on canola petals. Crop Protect. 26:100–107. 10.1016/j.cropro.2006.04.007

[10] Raio A Puopolo G Cimmino A Danti R Della RG Evidente A 2011 Biocontrol of cypress canker by the phenazine producer *Pseudomonas chlororaphis* subsp. *aureofaciens* strain M71. Biol. Contr. 58:133–138. 10.1016/j.biocontrol.2011.04.012

[11] Luo R Liu B Xie Y Li Z Huang W Yuan J He G Chen Y Pan Q Liu Y Tang J Wu G Zhang H Shi Y Liu Y Yu C Wang B Lu Y Han C Cheung DW Yiu SM Peng S Xiaoqian Z Liu G Liao X Li Y Yang H Wang J Lam TW Wang J 2012 SOAPdenovo2: an empirically improved memory-efficient short-read *de novo* assembler. GigaScience 1:18. 10.1186/2047-217X-1-18 23587118 PMC3626529

[12] Aziz RK Bartels D Best AA DeJongh M Disz T Edwards RA Formsma K Gerdes S Glass EM Kubal M Meyer F Olsen GJ Olson R Osterman AL Overbeek RA McNeil LK Paarmann D Paczian T Parrello B Pusch GD Reich C Stevens R Vassieva O Vonstein V Wilke A Zagnitko O 2008 The RAST server: rapid annotations using subsystems technology. BMC Genomics 9:75. 10.1186/1471-2164-9-75 18261238 PMC2265698

[13] Liu HM Dong DX Peng HS Zhang XH Xu YQ 2008 Phenazine-1-carboxylic acid biosynthesis in *Pseudomonas chlororaphis* GP72 is positively regulated by the sigma factor RpoN. World J. Microbiol. Biotechnol. 24:1961–1966. 10.1007/s11274-008-9655-0

[14] Mavrodi DV Ksenzenko VN Bonsall RF Cook RJ Boronin AM Thomashow LS 1998 A seven-gene locus for synthesis of phenazine-1-carboxylic acid by *Pseudomonas fluorescens* 2–79. J. Bacteriol. 180:2541–2548 9573209 10.1128/jb.180.9.2541-2548.1998 PMC107199

[15] Mavrodi DV Blankenfeldt W Thomashow LS 2006 Phenazine compounds in fluorescent *Pseudomonas* spp. biosynthesis and regulation. Annu. Rev. Phytopathol. 44:417–445. 10.1146/annurev.phyto.44.013106.145710 16719720

[16] Morohoshi T Wang WZ Suto T Saito Y Ito S Someya N Ikeda T 2013 Phenazine antibiotic production and antifungal activity are regulated by multiple quorum-sensing systems in *Pseudomonas chlororaphis* subsp. *aurantiaca* StFRB508. J. Biosci. Bioeng. 116:580–584. 10.1016/j.jbiosc.2013.04.022 23727350

[17] Hammer PE Burd W Hill DS Ligon JM van Pée KH 1999 Conservation of the pyrrolnitrin biosynthetic gene cluster among six pyrrolnitrin-producing strains. FEMS Microbiol. Lett. 180:39–44. 10.1111/j.1574-6968.1999.tb08775.x 10547442

[18] Keum YS Zhu YZ Kim JH 2011 Structure-inhibitory activity relationships of pyrrolnitrin analogues on its biosynthesis. Appl. Microbiol. Biotechnol. 89(:781–789. 10.1007/s00253-010-2872-0 20865257

[19] Laville J Blumer C Schroetter C Gaia V Défago G Keel C Haas D 1998 Characterization of the *hcnABC* gene cluster encoding hydrogen cyanide synthase and anaerobic regulation by ANR in the strictly aerobic biocontrol agent *Pseudomonas fluorescens* CHA0. J. Bacteriol. 180:3187–3196 9620970 10.1128/jb.180.12.3187-3196.1998 PMC107821

[20] Péchy-Tarr M Bruck DJ Maurhofer M Fischer E Vogne C Henkels MD Donahue KM Grunder J Loper JE Keel C 2008 Molecular analysis of a novel gene cluster encoding an insect toxin in plant-associated strains of *Pseudomonas fluorescens*. Environ. Microbiol. 10:2368–2386. 10.1111/j.1462-2920.2008.01662.x 18484997

[21] Lamont IL Martin LW 2003 Identification and characterization of novel pyoverdine synthesis genes in *Pseudomonas aeruginosa*. Microbiology 149 (Pt 4):833–842. 10.1099/mic.0.26085-0 12686626

[22] Owen JG Ackerley DF 2011 Characterization of pyoverdine and achromobactin in *Pseudomonas syringae* pv. *phaseolicola* 1448a. BMC Microbiol. 11:218. 10.1186/1471-2180-11-218 21967163 PMC3207962

[23] Shen X Chen M Hu H Wang W Peng H Xu P Zhang X 2012 Genome sequence of *Pseudomonas chlororaphis* GP72, a Root-Colonizing Biocontrol Strain. J. Bacteriol. 194:1269–1270. 10.1128/JB.06713-11 22328763 PMC3294805

[24] Xie K Peng H Hu H Wang W Zhang X 2013 OxyR, an important oxidative stress regulator to phenazines production and hydrogen peroxide resistance in *Pseudomonas chlororaphis* GP72. Microbiol. Res. 168:646–653. 10.1016/j.micres.2013.05.001 23778235

[25] Toyama H Nishibayashi E Saeki M Adachi O Matsushita K 2007 Factors required for the catalytic reaction of PqqC/D which produces pyrroloquinoline quinone. Biochem. Biophys. Res. Commun. 354:290–295. 10.1016/j.bbrc.2007.01.001 17223081

[26] Xiong XH Han S Wang JH Jiang ZH Chen W Jia N Wei HL Cheng H Yang YX Zhu B You S He JY Hou W Chen MX Yu CJ Jiao YH Zhang WC 2011 Complete genome sequence of the bacterium *Ketogulonicigenium vulgare* Y25. J. Bacteriol. 193:315–316. 10.1128/JB.01189-10 21037005 PMC3019938

[27] Choi O Kim J Kim JG Jeong Y Moon JS Park CS Hwang I 2008 Pyrroloquinoline quinone is a plant growth promotion factor produced by *Pseudomonas fluorescens* B16. Plant Physiol. 146:657–668. 10.1104/pp.107.112748 18055583 PMC2245851

